# Chronic Gastritis Is Associated with a Decreased High-Density Lipid Level: Histological Features of Gastritis Based on the Updated Sydney System

**DOI:** 10.3390/jcm9061856

**Published:** 2020-06-14

**Authors:** Dong-Hoon Kim, Byoung Kwan Son, Kyueng-Whan Min, Sang Kuk Han, Ji Ung Na, Pil Cho Choi, Hack-Lyoung Kim, Mi Jung Kwon, Young Ha Oh, Woon Yong Jung, Ji-Yong Moon, Sangmo Hong, Ki-Wook Oh, Young Seo Kim

**Affiliations:** 1 Departments of Pathology, Kangbuk Samsung Hospital, Sungkyunkwan University School of Medicine, Seoul 03181, Korea; idavid.kim@samsung.com; 2 Department of Internal Medicine, Eulji Hospital, Eulji University School of Medicine, Seoul 01830, Korea; sbk1026@eulji.ac.kr; 3 Department of Pathology, Hanyang University Guri Hospital, Hanyang University College of Medicine, Guri-si, Gyeonggi-do 11923, Korea; yhoh@hanyang.ac.kr (Y.H.O.); pathjwy@gmail.com (W.Y.J.); 4 Departments of Emergency Medicine, Kangbuk Samsung Hospital, Sungkyunkwan University School of Medicine, Seoul 03181, Korea; jiung.na@samsung.com (J.U.N.); pcmd.choi@samsung.com (P.C.C.); 5 Division of Cardiology, Department of Internal Medicine, Seoul National University College of Medicine, Seoul National University Boramae Medical Center, Seoul 07061, Korea; khl2876@gmail.com; 6 Department of Pathology, Hallym University Sacred Heart Hospital, Hallym University College of Medicine, Anyang-si, Gyeonggi-do 14068, Korea; mulank@hanmail.net; 7 Department of Internal Medicine, Hanyang University Guri Hospital, Hanyang University College of Medicine, Guri-si, Gyeonggi-do 11923, Korea; moonji@hanyang.ac.kr; 8 Division of Endocrinology, Department of Internal Medicine, Hanyang University Guri Hospital, Hanyang University College of Medicine, Guri-si, Gyeonggi-do 11923, Korea; lanugo@hanyang.ac.kr; 9 Department of Neurology, Hanyang University College of Medicine, Seoul 04763, Korea; kiwook-oh@hanyang.ac.kr (K.-W.O.); aescula@hanmail.net (Y.S.K.)

**Keywords:** *Helicobacter*, cholesterol, gastritis, cardiovascular diseases

## Abstract

Chronic gastritis could activate a systemic inflammatory response that could result in adverse lipid profiles. To determine the severity of chronic gastritis, *Helicobacter pylori* (HP), mononuclear cell (lymphocytes and plasma cells), and neutrophil scores were assessed on the basis of the updated Sydney system (USS), which is widely used for histological grading. The aim of this study was to assess the relationships between gastric histological features and lipid profile levels. This study included 15,322 males and 5929 females who underwent a health checkup and gastric biopsy at the Kangbuk Samsung Medical Center (KBSMC). We analyzed whether the HP, mononuclear cell, and neutrophil grades according to the USS were related to serum leukocyte count, unhealthy behaviors, and lipid profile levels. Gastritis with HP, neutrophils, or moderate to severe mononuclear cells was associated with an elevated serum leukocyte count. A high leukocyte count was related to increased low-density lipoproteins (LDL) and triglycerides/very-low-density lipoprotein (VLDL) and decreased high-density lipoproteins (HDL). In multivariate analyses, chronic gastritis with HP or moderate to severe mononuclear cells was significantly associated with decreased HDL in males, while mononuclear cells were significantly related to decreased HDL in females. Chronic gastritis was associated with an increased systemic inflammatory response, which was associated with unfavorable lipid profiles, especially low HDL levels.

## 1. Introduction

Dyslipidemia, such as increased low-density lipoprotein (LDL), very-low-density lipoprotein (VLDL), and triglyceride (TG) levels and decreased high-density lipoprotein (HDL) levels, is associated with a high incidence of metabolic syndrome and cardiovascular disease (CVD) [1,2]. Previous studies have demonstrated that lipid metabolism can be affected by alcohol consumption, diabetes, and hormonal imbalance as well as systemic inflammation and neoplasms [3,4]. Moreover, systemic inflammation with hypercholesterolemia can play an important role in the development of atherosclerosis [5].

*Helicobacter pylori* (HP) is a Gram-negative bacterium that colonizes the acidic gastric mucosa and causes chronic gastritis, ulcers, and cancer [6]. HP induces the recruitment of inflammatory cells such as neutrophils and mononuclear cells (lymphocytes and plasma cells) to the gastric mucosa [7]. Increased mononuclear cells in response to HP infection could induce the secretion of proinflammatory cytokines, leading to systemic inflammation such as elevated serum leukocyte count [8]. Another study suggested that the crosstalk between systemic inflammation and lipid metabolism has some effect on the alteration of lipids in the context of chronic gastritis [9]. Another study reported that HP was related to elevated levels of total cholesterol, LDL, lipoprotein(a), apolipoprotein B, and TGs, and decreased levels of HDL and apolipoprotein A-1 in the blood [10,11].

The updated Sydney system (USS), known as an objective grading system, is widely used to evaluate the severity of gastritis and includes the following elements: HP, neutrophils, mononuclear cells, atrophy, and intestinal metaplasia [12]. The USS scores are based on histological findings of gastric mucosal tissue. The USS is a more valuable grading system because it can represent not only the grade of HP but also that of neutrophils and mononuclear cells. Thus, each element of the USS can be used to quantitatively assess the effect of gastritis on lipid metabolism.

This study was conducted with people who underwent a large-scale gastroscopic biopsy and health checkup and is the first study to assess relationships between the histological features of gastritis and serum lipid profile levels. There have been several studies conducted on HP and lipid profiles, and the results of these studies have the following limitations: A small number of cases, analyses of relationships between carbon-13 urea (urea breath test) and cytotoxin-associated gene A (CagA), the use of indirect markers of HP and lipid profiles, and the use of serum samples [13,14].

The purpose of this study was to investigate whether the histological grading of local gastritis based on the USS could affect systemic homeostasis, such as lipid profiles and leukocyte levels in serum, or be associated with harmful lifestyle habits in a total of 21,251 people, including 15,322 males and 5929 females.

## 2. Materials and Methods

### 2.1. Patient Selection

We recruited 21,600 individuals who underwent a gastroscopy and histological examination during their health checkup (workplace health checkups) at the Kangbuk Samsung Medical Center (KBSMC) from 1 August 2006 to 30 September 2009 (excluding patients aged younger than 18 years). Patients with >2 mm-sized specimens and known clinical and laboratory parameters were included. A total of 349 patients were excluded based on the following criteria: (1) People who had a history of HP eradication or gastric surgery; (2) those who had already received anti-cholesterol therapy; (3) those suffering from severe liver disease, severe kidney disease, or cancer; and (4) those who had inadequate clinical and laboratory data. A total of 21,251 patients were included in the study. Gastric mucosal tissues from gastroscopic biopsy were assessed based on the USS [12].

### 2.2. Data Collection

The clinical and laboratory data collected from patients’ medical records included underlying diseases, medical history, exercise habits, and alcohol consumption. Systolic/diastolic blood pressure (SBP/DBP), body weight, and height were measured by a trained nurse. The body mass index (BMI) was calculated by dividing the body weight in kilograms (kg) by height in meters squared (m^2^). Regular exercise was defined as exercising for a minimum of 30 min three days a week. Alcohol consumption was defined as the consumption of alcohol at least once per week. Hypertension was defined as an SBP of ≥140 mmHg, a DBP of ≥90 mmHg, or a history of anti-hypertensive therapy. Serum biochemical profiles were measured, including total cholesterol, LDL, VLDL, TGs, HDL, and serum leukocyte count.

The Laboratory Medicine Department at KBSMC in Seoul, Korea, is accredited by the Korean Society of Laboratory Medicine and the Korean Association of Quality Assurance for Clinical Laboratories. The laboratory participated in the College of American Pathologists Survey Proficiency Testing.

### 2.3. Microscopic Examination for Updated Sydney System Scoring

**Figure 1:**
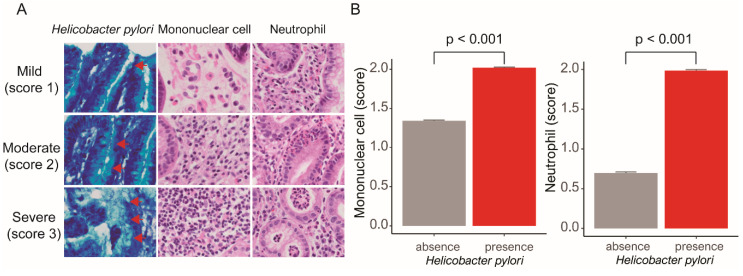
(**A**) Mild (score 1), moderate (score 2), and severe (score 3) *Helicobacter pylori* (red arrowhead) (Giemsa, original magnification ×400), mononuclear cells (hematoxylin and eosin, original magnification ×100), and neutrophils (hematoxylin and eosin, original magnification ×100) (**B**) The presence of *Helicobacter pylori* is associated with higher mononuclear cell (right) and neutrophil (left) counts than the absence of *Helicobacter pylori* (mononuclear cells: 1.34 and 2.02, respectively, *p* < 0.001; neutrophils: 0.7 and 1.99, respectively, *p* < 0.001).

The specimens were fixed in 10% formalin, embedded in paraffin on the oriented edge, and cut into 4-µm-thick sections. All sections were stained with hematoxylin-eosin and Giemsa stain for histopathological evaluation. We assessed histological features of gastritis in gastric mucosa using a light microscope in a blinded fashion. According to the USS based on HP, neutrophils, mononuclear cells, atrophy, and intestinal metaplasia, we evaluated the following three elements: HP, mononuclear cells, and neutrophils [12]. The density of each element was recorded separately as follows: 0 (absence), 1 (mild), 2 (moderate), and 3 (severe) (Figure 1A). The grading of HP, mononuclear cells, and neutrophils is based on the highest possible density if two or more biopsy tissues are available (HP: Absence, 10,361 (48.8%); mild, 4438 (20.9%); moderate, 4532 (21.3%); severe, 1920 (9%)) (mononuclear cell: Absence, 336 (1.6%); mild, 7282 (34.2%); moderate, 12,286 (57.8%); severe, 1347 (6.3%)) (neutrophil: Absence, 5556 (26.1%); mild, 4929 (23.2%); moderate, 8388 (39.5%); severe, 2378 (11.2%)). HP, mononuclear cells, and neutrophils were each divided into two groups as follows: Presence of HP vs. absence of HP, absence of mononuclear cells and mild mononuclear cells vs. moderate/severe mononuclear cells, and presence of neutrophils vs. absence of neutrophils.

### 2.4. Statistical Analysis

Correlations between clinical laboratory parameters and grade of gastritis, including HP, mononuclear cells, and neutrophils, were analyzed using the chi-square test, Student’s *t*-test, and Pearson correlation coefficient. Coefficients between gastritis and lipid profile levels, such as LDL, TGs, LDL and VLDL, were tested with multiple binary logistic analyses after adjustment for the following confounders: Age, body mass index (BMI), smoking, exercise rate, and alcohol consumption; *p*-values less than 0.05 were considered statistically significant. Statistical analyses were conducted using IBM SPSS Statistics for Windows, version 24.0 (IBM, Corp., Armonk, NY, USA) and R package.

## 3. Results

### 3.1. Clinical and Laboratory Parameters According to Helicobacter Pylori, Mononuclear Cells, and Neutrophils

HP was associated with increased mononuclear cells and neutrophils (mononuclear cell score, 1.34 and 2.02, respectively, *p* < 0.001; neutrophil score, 0.7 and 1.99, respectively, *p* < 0.001) (Figure 1B). Mononuclear cells were associated with high SBP and DBP (*p* < 0.001 and *p* = 0.001, respectively).

Among the 10,361 people without HP, moderate/severe mononuclear cells were associated with high BMI, smoking, alcohol consumption, high SBP/DBP, and increased leukocyte count in the male group (all *p* < 0.05). Neutrophil was related to old age and increased leukocyte count in the male group (all *p* < 0.05). In the female group, moderate/severe mononuclear cells were associated with old age, high BMI, high SBP, and increased leukocyte count (all *p* < 0.05). Neutrophil was related to old age, high BMI, smoking, and high SBP/DBP in the female group (all *p* < 0.05). There were significant relationships between moderate/severe mononuclear cells and unfavorable lipid profile levels in the female group. (all *p* < 0.05).

In the 10,980 people with HP, moderate/severe mononuclear cells were associated with young age, diabetes, and high SBP in the male group (all *p* < 0.05). There was a relationship between neutrophil and young age in the male and female groups (all *p* < 0.05). Moderate/severe mononuclear cells were associated with young age and low HDL in the female group (all *p* < 0.05) (Table 1).

In the 15,322 males, HP, neutrophils, and moderate/severe mononuclear cells were associated with young age, smoking, and increased leukocyte count (all *p* < 0.05). HP and moderate/severe mononuclear cells were related to low exercise rate and alcohol intake (all *p* < 0.05). VLDL and TGs were increased in individuals with neutrophils and moderate/severe mononuclear cells (all *p* < 0.05). Among the 5929 females, HP, neutrophils, and moderate/severe mononuclear cells were associated with increased leukocyte counts (all *p* < 0.001). Neutrophils and moderate/severe mononuclear cells were related to high BMI and smoking (all *p* < 0.05). LDL was increased while HDL was decreased in moderate/severe mononuclear cells (all *p* < 0.05) (Table 2).

In multivariate analyses, after adjustment for confounders such as age, BMI, smoking, exercise rate, and alcohol consumption, gastritis with HP or mononuclear cells was significantly associated with low HDL levels in males (all *p* < 0.05). In females, low HDL was statistically related to gastritis with mononuclear cells (all *p* < 0.05) (Table 3).

### 3.2. Associations among Helicobacter Pylori, Mononuclear Cells, Neutrophils, Serum Leukocyte Count and Lipid Profiles

**Figure 2:**
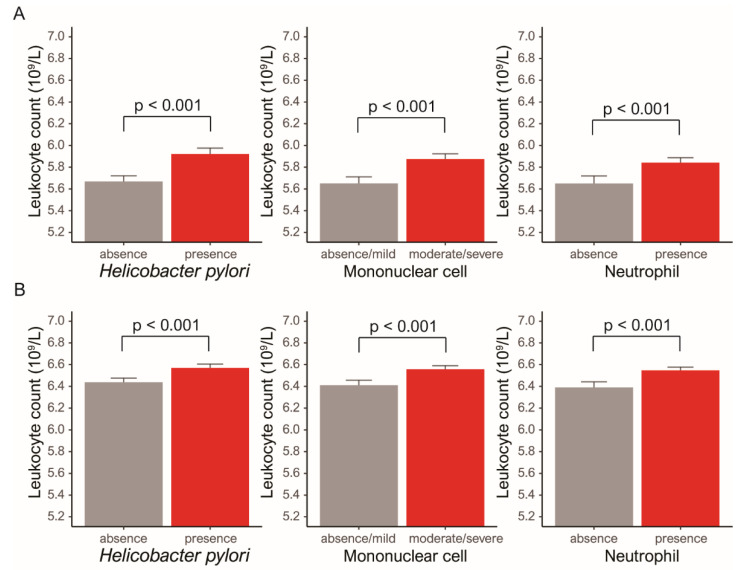
(**A**) In the female group, the serum leukocyte count was increased in the presence of *Helicobacter pylori* (absence, 5.66 versus presence, 5.92 (10^9^/L)]), mononuclear cells (absence/mild, 5.65 versus moderate/severe 5.82 (10^9^/L)), and neutrophils (absence, 5.65 versus presence, 5.84 (10^9^/L)). (**B**) In the male group, the serum leukocyte count was increased in the presence of *Helicobacter pylori* (absence, 6.44 versus presence, 6.57 (10^9^/L)), mononuclear cells (absence/mild, 6.41 versus moderate severe 6.56 (10^9^/L)), and neutrophils (absence, 6.39 versus presence, 6.55 (10^9^/L)) (error bars: standard errors of the mean).

**Figure 3:**
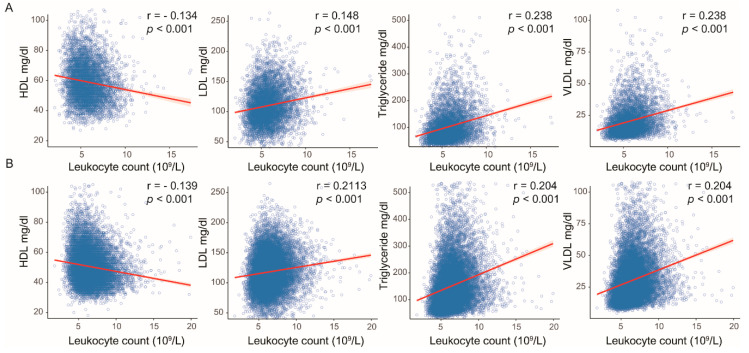
(**A**) In the female group, a high serum leukocyte count was associated with decreased high-density lipoprotein (HDL) but increased low-density lipoprotein (LDL), triglycerides and very-low-density lipoprotein (VLDL) (HDL, *r* = −0.134; LDL, *r* = 0.148; triglycerides, *r* = 0.238; VLDL, *r* = 0.238, all *p* < 0.001). (**B**) In the male group, a high serum leukocyte count was associated with decreased high-density lipoprotein (HDL) but increased low-density lipoprotein (LDL), triglycerides and very-low-density lipoprotein (VLDL) (HDL, *r* = - 0.139; LDL, *r* = 0.113; triglycerides, *r* = 0.204; VLDL, *r* = 0.204, all *p* < 0.001).

We explored whether serum leukocyte counts reflected systemic inflammatory effects on HP, mononuclear cells, and neutrophils and their subsequent effects on serum lipid profiles. First, we analyzed the relationships between the grade of HP, mononuclear cells, neutrophils, and serum leukocyte count. In the analyses of the female group and the male group, a high serum leukocyte count was found to be elevated in individuals with gastritis associated with HP, neutrophils and mononuclear cells (female: HP (absence vs. presence), 5.66 (10^9^/L) vs. 5.92; mononuclear cells (absence/mild vs. moderate/severe), 5.65 vs. 5.87; neutrophils (absence vs. presence), 5.65 vs. 5.84, male: HP, 6.44 vs. 6.57; mononuclear cells, 6.41 vs. 6.56; neutrophils, 6.39 vs. 6.55, all *p* < 0.001) (Figure 2). Second, we analyzed the relationships between serum leukocyte counts and lipid levels. In the analyses of the female group and the male group, the Pearson coefficient showed good correlation between high leukocyte count and lipid levels (female: HDL, *r* = −0.134; LDL, *r* = 0.148; triglycerides, *r* = 0.238; VLDL, *r* = 0.238, male: HDL, *r* = −0.139; LDL, *r* = 0.113; triglyceride, *r* = 0.204; VLDL, *r* = 0.204; all *p* < 0.001) (Figure 3), suggesting that systemic inflammation had a significant effect on lipid metabolism These results demonstrated that the severity of gastritis could be associated with elevated lipid profile levels.

## 4. Discussion

In our study, gastritis with HP, neutrophils, and mononuclear cells was associated with unfavorable lipid profile levels, including increased LDL/VLDL/TGs and decreased HDL, as well as unhealthy behaviors, such as decreased exercise rate, history of smoking, and increased alcohol intake. In people without HP, neutrophils and mononuclear cells were related to unfavorable lipid profile levels, whereas in people with HP, there was a correlation between mononuclear cells and low HDL. We found that mononuclear cell and neutrophilic infiltration independent of HP could affect lipid metabolism in people with gastritis. Chronic gastritis was associated with a high serum leukocyte count representing an increased systemic inflammatory response in the female and the male groups. Interestingly, there was a positive correlation between high leukocyte count and increased LDL/VLDL/TGs and decreased HDL. These results demonstrated that histological features of gastritis could be associated with elevated lipid profile levels. In multivariate analyses, chronic gastritis with mononuclear cells was significantly related to low HDL in both the male and female groups, while chronic gastritis with HP was significantly associated with low HDL in the male group.

Epidemiological surveys have indicated that the possible pathological consequences of HP infection are not restricted to the gastroduodenal tract [15,16,17]. *Helicobacter* gastritis is correlated with a wide range of extradigestive diseases, such as heart, vessel, skin, endocrine, respiratory, hematopoietic, immune, and central nervous system conditions [18,19]. CVD risk was more closely related to HP infection than to other clinical diseases. Previous studies have reported a relationship between HP and atherosclerosis, which is explained by the immune cross-reactivity between CagA (HP virulence factor) and the arterial wall [20,21]. CagA could enhance immune-related cytokine production, which increases systemic inflammatory responses such as increased leukocyte counts [22]. Indeed, higher leukocyte counts are associated with an increased incidence of CVD, stroke, and sudden death [23].

USS is accepted worldwide as the gold standard for the histopathological assessment of gastritis. USS scoring is based on five elements, namely, HP, chronicity (monocytic infiltration), activity (neutrophilic infiltration), glandular atrophy, and intestinal metaplasia. Published data described that HP and inflammatory cell infiltration increase CVD risk [24]. The assessment of the severity of gastritis based on HP, mononuclear cells, and neutrophils should be focused not only on local inflammation but also on the whole body. Previous studies suggested that HP was related to unfavorable lipid profile levels, hypercoagulation, and endothelial dysfunction as a result of high homocysteine levels, an increase in vasoconstrict factors and the stimulation of platelet aggregation and then the development of atherosclerotic plaques through the action of reactive oxygen species and circulating lipid peroxides [25,26,27]. The accumulation of lipids within the arterial wall in the context of the HP infection-related systemic inflammatory response could lead to the development of atherosclerosis with vessel wall injury and may contribute to increased susceptibility to myocardial infarction [28,29,30]. A high serum leukocyte count associated with local gastritis could generate inflammatory mediators, cytokines, and oxygen-free radicals, which result in cardiac damage [31]. A study by Takeshita et al. demonstrated that increased systemic inflammation could induce ischemic heart disease [32]. Our study revealed that chronic gastritis, similar to local inflammatory processes, plays an important role in high leukocyte count, increased LDL/VLDL/TGs, and decreased HDL. We suggest that HP-, neutrophil- and mononuclear cell-related chronic gastritis could promote systemic inflammation and unfavorable lipid metabolism over time, followed by CVD risk.

This study showed that chronic gastritis was significantly associated with unhealthy behavior, such as less exercise, smoking, alcohol intake, and high BMI. Previous studies have described that exercise-induced nitric oxide (NO) production could contribute to improvement in endothelial function and cardiovascular homeostasis [33,34]. Smoking and high BMI are well-known modifiable factors for CVD [35]. Other studies have demonstrated that weight gain following smoking cessation is associated with a potential risk for CVD [36,37], but large-scale studies showed that weight gain associated with smoking cessation did not outweigh the benefits in terms of CVD risk [38,39]. Regular alcohol intake could elevate blood pressure and is attributed to 16% of hypertension cases [40]. In several studies, blood pressure was reduced after alcohol reduction in patients with hypertension [40,41,42].

There are some limitations that should be acknowledged in this study. This study analyzed subjects who underwent gastroscopy during their health checkups, and there is a potential for selection bias. The relationship among chronic gastritis, leukocyte count and lipid levels could not be conclusively proven due to the cross-sectional design of this study. Hence, longitudinal prospective studies should be conducted to identify the continuous associations among these factors over time. The people enrolled in this study had diverse lifestyle habits, such as exercise rate, smoking, and alcohol intake, which might worsen lipid metabolism. Published data reported that people who drink a large amount of alcohol or lack exercise usually suffer from chronic gastritis [43,44]. In the present study, several factors associated with chronic gastritis and poor lipid profiles overlapped. Hence, it was difficult to make accurate conclusions. To complement these points, we adjusted for potential confounders, including age, sex, BMI, smoking, exercise rate, and alcohol consumption, to clarify the relationship between chronic gastritis and low HDL level in both male and female groups; however, the relationship between these variables remains unclear.

In summary, this large-scale cohort study demonstrated that histological features such as HP infection and monocytic infiltration were associated with increased LDL/VLDL/TGs and decreased HDL as well as elevated leukocyte count in chronic gastritis patients. In multivariate analyses in the female group and the male group, chronic gastritis with mononuclear cells was significantly related to decreased HDL. In the male group, HP infection was also associated with low HDL. Chronic gastritis could be an important factor in the complex mechanisms involved in lipid metabolism. In the future, experimental studies are necessary to clarify the relationships between chronic gastritis and abnormal lipid profiles.

## Figures and Tables

**Table 1:** Comparison of clinical laboratory parameters according to mononuclear cells and neutrophils in 10,361 people without *Helicobacter pylori* and 10,890 people with *Helicobacter pylori* using updated Sydney system.

Absence of *Helicobacter pylori* (*N* = 10,361)												
Parameters	Male (*n* = 15,322)						Female (*n* = 5929)					
	Mononuclear cell			Neutrophil			Mononuclear cell			Neutrophil		
	Absence/ Mild (*n* = 4511)	Moderate/ Severe (*n* = 2671)	*p*	Absence (*n* = 3459)	Presence (*n* = 3723)	*p*	Absence/ Mild (*n* = 2087)	Moderate/ Severe (*n* = 1092)	*p*	Absence (*n* = 1610)	Presence (*n* = 1569)	*p*
Clinical												
Age, years	44.4 ± 9.8	44.7 ± 9.9	0.23 *	44.2 ± 9.6	44.7 ± 10	**0.041** *	44.5 ± 10.5	46.8 ± 11.2	**<0.001** *	44.5 ± 10.6	46.1 ± 11	**<0.001** *
Body mass index (kg/m^2^)	24.6 ± 2.8	24.7 ± 2.8	**0.009** *	24.6 ± 2.8	24.7 ± 2.8	0.076 *	22.8 ± 3	23.1 ± 3.3	**0.007** *	22.8 ± 3.1	23.1 ± 3.2	**0.002** *
^‡^ Regular exercise, %	1259 (27.9)	791 (29.6)	0.129 ^†^	954 (27.6)	1096 (29.4)	0.086 ^†^	552 (26.4)	297 (27.2)	0.681 ^†^	422 (26.2)	427 (27.2)	0.549 ^†^
Cigarette smoking, %	3428 (76)	2048 (76.7)	0.529 ^†^	2621 (75.8)	2855 (76.7)	0.379 ^†^	256 (12.3)	151 (13.8)	0.232 ^†^	184 (11.4)	223 (14.2)	**0.022 ^†^**
^§^ Alcohol consumption, %	3694 (81.9)	2236 (83.7)	**0.05** ^†^	2840 (82.1)	3090 (83.0)	0.334 ^†^	703 (33.7)	379 (34.7)	0.59 ^†^	563 (35)	519 (33.1)	0.277 ^†^
Hypertension, %	1111 (24.6)	678 (25.4)	0.492 ^†^	864 (25)	925 (24.8)	0.918 ^†^	355 (17)	182 (16.7)	0.845 ^†^	264 (16.4)	273 (17.4)	0.48 ^†^
Diabetes, %	257 (5.7)	150 (5.6)	0.927 ^†^	181 (5.2)	226 (6.1)	0.138 ^†^	75 (3.6)	39 (3.6)	0.999 ^†^	59 (3.7)	55 (3.5)	0.884 ^†^
Hepatitis, %	681 (10.3)	366 (9.7)	0.351 ^†^	489 (9.6)	558 (10.5)	0.138 ^†^	187 (9)	116 (10.6)	0.146 ^†^	139 (8.6)	164 (10.5)	0.092 ^†^
SBP, mmHg	117.5 ± 12.5	118.2 ± 12.7	**0.027** *	117.8 ± 12.7	117.7 ± 12.5	0.675 *	111.5 ± 13.6	112.9 ± 14.1	**0.006** *	111.1 ± 13.7	112.9 ± 13.9	**<0.001** *
DBP, mmHg	76.2 ± 8.6	76.7 ± 8.7	**0.013** *	76.4 ± 8.6	76.3±8.7	0.677 *	72.3 ± 9.4	73.0 ± 9.2	0.072 *	72.2 ± 9.3	73.0 ± 9.4	**0.018** *
Laboratory												
HDL, mg/dL	50.9 ± 11.2	50.5 ± 11	0.129 *	50.8 ± 11.3	50.7 ± 11.1	0.8 *	59.5 ± 13.6	58.2 ± 12.8	**0.008** *	59.2 ± 13.4	58.8 ± 13.3	0.378 *
LDL, mg/dL	117.8 ± 30.0	118.8 ± 29.6	0.183 *	118.0 ± 29.6	118.4 ± 30.1	0.557 *	108.6 ± 31.2	111.5 ± 30.6	**0.012** *	109.8 ± 31.1	109.4 ± 30.9	0.768 *
Triglycerides, mg/dL	148.9 ± 0.9	152.9 ± 93.5	0.073 *	148.8 ± 89.2	151.8 ± 94.3	0.176 *	101.9 ± 61.5	104.9 ± 61.5	0.2 *	102.4 ± 60.2	103.5 ± 62.8	0.606 *
VLDL, mg/dL	29.8 ± 18.2	30.6 ± 18.7	0.073 *	29.8 ± 17.8	30.4 ± 18.9	0.176 *	20.4 ± 12.3	21.0 ± 12.3	0.2 *	20.5 ± 12.0	20.7 ± 12.6	0.606 *
Leukocyte count (10^9^/L)	6.4 ± 1.7	6.5 ± 1.7	**<0.001** *	6.4 ± 1.6	6.5 ± 1.7	**0.001** *	5.6 ± 1.5	5.7 ± 1.5	**0.04** *	5.6 ± 1.5	5.7 ± 1.5	0.254 *
Presence of *Helicobacter pylori* (*N* = 10,890)												
Parameters	Male (*n* = 15,322)						Female (*n* = 5929)					
	Mononuclear cell			Neutrophil			Mononuclear cell			Neutrophil		
	Absence/ Mild (*n* = 740)	Moderate/ Severe (*n* = 7400)	*p*	Absence (*n* = 352)	Presence (*n* = 7788)	*p*	Absence/ Mild (*n* = 280)	Moderate/ Severe (*n* = 2470)	*p*	Absence (*n* = 135)	Presence (*n* = 2615)	*p*
Clinical												
Age, years	43.8 ± 9.2	42.2 ± 8.6	**<0.001** *	44.3 ± 9.2	42.3 ± 8.6	**<0.001** *	46.1 ± 10.2	44.3 ± 9.8	**0.003** *	46.1 ± 10.5	44.4 ± 9.8	**0.042** *
Body mass index (kg/m^2^)	24.8 ± 2.8	24.7 ± 2.8	0.076 *	24.7 ± 2.7	24.7 ± 2.8	0.974 *	22.8 ± 2.9	23.0 ± 3.1	0.461 *	23.0 ± 2.8	23.0 ± 3.1	0.922 *
^‡^Regular exercise, %	202 (27.3)	1877 (25.4)	0.269 ^†^	89 (25.3)	1990 (25.6)	0.96 ^†^	76 (27.1)	651 (26.4)	0.833 ^†^	34 (25.2)	693 (26.5)	0.812 ^†^
Cigarette smoking, %	570 (77)	5795 (78.3)	0.447 ^†^	267 (75.9)	6098 (78.3)	0.307 ^†^	29 (10.4)	358 (14.5)	0.073 ^†^	14 (10.4)	373 (14.3)	0.254 ^†^
^§^Alcohol consumption, %	631 (85.3)	6298 (85.1)	0.949 ^†^	296 (84.1)	6633 (85.2)	0.631 ^†^	108 (38.6)	922 (37.3)	0.732 ^†^	59 (43.7)	971 (37.1)	0.148 ^†^
Hypertension, %	161 (21.8)	1657 (22.4)	0.727 ^†^	94 (26.7)	1724 (22.1)	0.05 ^†^	45 (16.1)	395 (16)	0.999 ^†^	28 (20.7)	412 (15.8)	0.155 ^†^
Diabetes, %	53 (7.2)	365 (4.9)	**0.011 ^†^**	22 (6.2)	396 (5.1)	0.398 ^†^	5 (1.8)	84 (3.4)	0.204 ^†^	3 (2.2)	86 (3.3)	0.665 ^†^
Hepatitis, %	89 (12)	772 (10.4)	0.2 ^†^	36 (10.2)	825 (10.6)	0.897 ^†^	27 (9.6)	247 (10)	0.933 ^†^	13 (9.6)	261 (10)	0.999 ^†^
SBP, mmHg	116.4 ± 12.6	117.6 ± 12.1	**0.01** *	117.1 ± 13.3	117.5 ± 12.1	0.584 *	111.3 ± 13.9	111.7 ± 13.8	0.647 *	111.9 ± 15.7	111.7 ± 13.7	0.908 *
DBP, mmHg	76.3 ± 8.2	76.4 ± 8.6	0.814 *	77.0 ± 9.1	76.4 ± 8.5	0.191 *	72.1 ± 9.6	72.0 ± 9.4	0.955 *	72.0 ± 10.2	72.0 ± 9.4	0.954 *
Laboratory												
HDL, mg/dL	51.0 ± 11.1	50.3 ± 10.7	0.084 *	50.0 ± 11.0	50.3 ± 10.8	0.598 *	61.2 ± 14.5	58.9 ± 12.9	**0.012** *	61.0 ± 13.4	59.0 ± 13	0.076 *
LDL, mg/dL	118.2 ± 29.9	118.6 ± 29.8	0.72 *	116.6 ± 27.3	118.7 ± 29.9	0.172 *	110.7 ± 29.2	110.0 ± 30.6	1.726 *	111.8 ± 29.7	110.0 ± 30.5	1.499 *
Triglycerides, mg/dL	154.9 ± 101.8	153.1 ± 96.4	0.66 *	152.8 ± 102.3	153.3 ± 96.6	0.918 *	103.0 ± 70.9	102.5 ± 60	0.914 *	100.5 ± 63.4	102.7 ± 61.1	0.692 *
VLDL, mg/dL	31.0 ± 20.4	30.6 ± 19.3	0.66 *	30.6 ± 20.5	30.7 ± 19.3	0.918 *	20.6 ± 14.2	20.5 ± 12.0	0.914 *	20.1 ± 12.7	20.5 ± 12.2	0.692 *
Leukocyte count (10^9^/L)	51.0 ± 11.1	50.3 ± 10.7	0.084 *	50.0 ± 11.0	50.3 ± 10.8	0.598 *	5.8 ± 1.4	5.9 ± 1.5	0.206 *	5.8 ± 1.5	5.9 ± 1.5	0.278 *

SBP, systolic blood pressure; DBP, diastolic blood pressure; HDL, high-density lipoprotein; LDL, low-density lipoprotein; VLDL, very-low-density lipoprotein; Values are expressed as the mean ± standard deviation or number. * Student’s *t*-test; ^†^ Chi-square test; ^‡^ Regular exercise is defined as exercising 3 or more days per week; ^§^ Alcohol consumption is defined as consuming alcohol at least once per week; *p* < 0.05 is shown in bold.

**Table 2:** Comparison of clinicolaboratory parameters according to *Helicobacter pylori*, mononuclear cells and neutrophils in the group of 15,322 males and the group of 5929 females using updated Sydney system.

Male (*n* = 15,322)									
Parameters	*Helicobacter pylori*			Mononuclear Cell			Neutrophil		
	Absence (*n* = 7182)	Presence (*n* = 8140)	*p*	Absence/Mild (*n* = 5251)	Moderate/Severe (*n* = 10,071)	*p*	Absence (*n* = 3811)	Presence (*n* = 11,511)	*p*
Clinical									
Age, years	44.5 ± 9.8	42.4 ± 8.7	**<0.001** *	44.3 ± 9.7	42.9 ± 9	**<0.001** *	44.3 ± 9.6	43.1 ± 9.2	**<0.001** *
Body mass index (kg/m^2^)	24.6 ± 2.8	24.7 ± 2.8	0.337 *	24.6 ± 2.8	24.7 ± 2.8	0.112 *	24.6 ± 2.8	24.7 ± 2.8	0.056 *
^‡^ Regular exercise	1905 (26.5)	1893 (23.3)	**<0.001** ^†^	1374 (26.2)	2424 (24.1)	**0.005** ^†^	985 (25.8)	2813 (24.4)	0.085 ^†^
Cigarette smoking	5476 (76.2)	6365 (78.2)	**0.004** ^†^	3998 (76.1)	7843 (77.9)	**0.016** ^†^	2888 (75.8)	8953 (77.8)	**0.011** ^†^
^§^ Alcohol consumption	5930 (82.6)	6929 (85.1)	**<0.001** ^†^	4325 (82.4)	8534 (84.7)	**<0.001** ^†^	3136 (82.3)	9723 (84.5)	0.002 ^†^
Hypertension	1789 (24.9)	1818 (22.3)	**<0.001** ^†^	1272 (24.2)	2335 (23.2)	0.156 ^†^	958 (25.1)	2649 (23)	0.008 ^†^
Diabetes	407 (5.7)	418 (5.1)	0.156 ^†^	310 (5.9)	515 (5.1)	**0.044** ^†^	203 (5.3)	622 (5.4)	0.888 ^†^
Hepatitis	744 (10.4)	861 (10.6)	0.679 ^†^	583 (11.1)	1022 (10.1)	0.071 ^†^	386 (10.1)	1219 (10.6)	0.438 ^†^
SBP, mmHg	117.7 ± 12.6	117.5 ± 12.2	0.205 *	117.3 ± 12.5	117.7 ± 12.3	**0.049** *	117.7 ± 12.8	117.6 ± 12.3	0.446 *
DBP, mmHg	76.3 ± 8.6	76.4 ± 8.6	0.695 *	76.2 ± 8.6	76.5 ± 8.6	**0.039** *	76.4 ± 8.6	76.4 ± 8.6	0.563 *
Laboratory									
HDL, mg/dL	50.7 ± 11.2	50.3 ± 10.8	**0.02** *	50.9 ± 11.2	50.3 ± 10.8	**0.002** *	50.7 ± 11.2	50.5 ± 10.9	0.234 *
LDL, mg/dL	118.2 ± 29.8	118.6 ± 29.8	0.408 *	117.9 ± 30	118.6 ± 29.7	0.12 *	117.8 ± 29.4	118.6 ± 30	0.189 *
Triglycerides, mg/dL	150.4 ± 91.9	153.3 ± 96.9	0.054 *	149.7 ± 92.5	153.1 ± 95.6	**0.035** *	149.2 ± 90.5	152.8 ± 95.9	**0.035** *
VLDL, mg/dL	30.1 ± 18.4	30.7 ± 19.4	0.054 *	29.9 ± 18.5	30.6 ± 19.1	**0.035** *	29.8 ± 18.1	30.6 ± 19.2	**0.035** *
Leukocyte count (10^9^/L)	6.4 ± 1.7	6.6 ± 1.6	**<0.001** *	6.4 ± 1.7	6.6 ± 1.6	**<0.001** *	6.4 ± 1.7	6.5 ± 1.6	**<0.001** *
**Female (*n* = 5929)**									
Parameters	*Helicobactor pylori*			Mononuclear cell			Neutrophil		
	Absence (*n* = 3179)	Presence (*n* = 2750)	*p*	Absence/Mild (*n* = 2367)	Moderate/Severe (*n* = 3562)	*p*	Absence (*n* = 1745)	Presence (*n* = 4184)	*p*
Clinical									
Age, years	45.3 ± 10.8	44.5 ± 9.8	**0.002** *	44.7 ± 10.5	45.0 ± 10.3	0.207 *	44.6 ± 10.6	45.0 ± 10.3	0.229 *
Body mass index (kg/m^2^)	22.9 ± 3.1	23.0 ± 3.1	0.704 *	22.8 ± 3	23.0 ± 3.2	**0.012** *	22.8 ± 3	23.0 ± 3.1	**0.007** *
^‡^ Regular exercise	807 (25.4)	694 (25.2)	0.919 ^†^	603 (25.5)	898 (25.2)	0.842 ^†^	436 (25)	1065 (25.5)	0.73 ^†^
Cigarette smoking	407 (12.8)	387 (14.1)	0.163 ^†^	285 (12.0)	509 (14.3)	**0.014** ^†^	198 (11.3)	596 (14.2)	**0.003** ^†^
^§^ Alcohol consumption	1082 (34.0)	1030 (37.5)	**0.007** ^†^	811 (34.3)	1301 (36.5)	0.08 ^†^	622 (35.6)	1490 (35.6)	0.999 ^†^
Hypertension	537 (16.9)	440 (16)	0.374 ^†^	400 (16.9)	577 (16.2)	0.499 ^†^	292 (16.7)	685 (16.4)	0.761 ^†^
Diabetes	114 (3.6)	89 (3.2)	0.505 ^†^	80 (3.4)	123 (3.5)	0.937 ^†^	62 (3.6)	141 (3.4)	0.783 ^†^
Hepatitis	303 (9.5)	274 (10)	0.606 ^†^	214 (9.0)	363 (10.2)	0.156 ^†^	152 (8.7)	425 (10.2)	0.096 ^†^
SBP, mmHg	112.0 ± 13.8	111.7 ± 13.8	0.401 *	111.5 ± 13.7	112.1 ± 13.9	0.095 *	111.2 ± 13.8	112.2 ± 13.8	**0.014** *
DBP, mmHg	72.6 ± 9.3	72.0 ± 9.4	**0.033** *	72.3 ± 9.4	72.3 ± 9.3	0.961 *	72.2 ± 9.3	72.4 ± 9.4	0.395 *
Laboratory									
HDL, mg/dL	59.0 ± 13.3	59.1 ± 13.1	0.856 *	59.7 ± 13.7	58.7 ± 12.8	**0.004** *	59.4 ± 13.4	58.9 ± 13.1	0.233 *
LDL, mg/dL	109.6 ± 31	110.1 ± 30.4	0.531 *	108.9 ± 30.9	110.5 ± 30.6	**0.044** *	109.9 ± 31.0	109.8 ± 30.6	0.888 *
Triglycerides, mg/dL	102.9 ± 61.5	102.6 ± 61.2	0.815 *	102.0 ± 62.6	103.2 ± 60.5	0.466 *	102.2 ± 60.5	103.0 ± 61.7	0.67 *
VLDL, mg/dL	20.6 ± 12.3	20.5 ± 12.2	0.815 *	20.4 ± 12.5	20.6 ± 12.1	0.466 *	20.4 ± 12.1	20.6 ± 12.3	0.67 *
Leukocyte count (10^9^/L)	5.7 ± 1.5	5.9 ± 1.5	**<0.001** *	5.7 ± 1.5	5.9 ± 1.5	**<0.001** *	5.6 ± 1.5	5.8 ± 1.5	**<0.001** *

BMI, body mass index; SBP, systolic blood pressure; DBP, diastolic blood pressure; HDL, high-density lipoprotein; LDL, low-density lipoprotein; VLDL, very-low-density lipoprotein. Values are expressed as the mean ± standard deviation or number. * Student’s *t*-test; ^†^ Chi-square test; ^‡^ Regular exercise is defined as exercising 3 or more days per week; ^§^ Alcohol consumption is defined as consuming alcohol at least once per week; *p* < 0.05 is shown in bold.

**Table 3:** Multivariate binary logistic analyses of LDL, VLDL, triglycerides and HDL according to *Helicobacter pylori*, mononuclear cells and neutrophils using updated Sydney system (USS).

Parameters		Male (*n* = 15,322)				Female (*n* = 5929)			
		Odds Ratio	95% CI		*p*	Odds Ratio	95% CI		*p*
			Low	High			Low	High	
HDL (<60 mg/dL)	HP								
	Crude	0.892	0.822	0.968	**0.006**	0.966	0.871	1.07	0.505
	*Adjusted	0.898	0.826	0.977	**0.012**	0.926	0.832	1.029	0.154
	Mononuclear cell								
	Crude	0.904	0.83	0.984	**0.02**	0.891	0.802	0.989	**0.03**
	*Adjusted	0.913	0.837	0.997	**0.042**	0.888	0.798	0.99	**0.032**
	Neutrophil								
	Crude	0.942	0.858	1.034	0.21	0.93	0.831	1.041	0.206
	*Adjusted	0.95	0.864	1.045	0.295	0.946	0.843	1.063	0.35
LDL (>130 mg/dL)	HP								
	Crude	1.027	0.96	1.098	0.44	1.07	0.949	1.207	0.267
	*Adjusted	1.026	0.958	1.098	0.462	1.141	1.007	1.292	**0.038**
	Mononuclear cell								
	Crude	1.043	0.972	1.12	0.238	1.088	0.962	1.23	0.179
	*Adjusted	1.037	0.966	1.114	0.314	1.072	0.944	1.217	0.283
	Neutrophil								
	Crude	1.05	0.971	1.135	0.22	0.999	0.876	1.139	0.987
	*Adjusted	1.044	0.965	1.129	0.28	0.973	0.849	1.115	0.692
Triglycerides (>150 mg/dL)	HP								
	Crude	1.06	0.993	1.131	0.081	1.043	0.905	1.203	0.56
	*Adjusted	1.039	0.971	1.112	0.265	1.134	0.977	1.317	0.098
	Mononuclear cell								
	Crude	1.058	0.988	1.133	0.108	1.11	0.959	1.284	0.162
	*Adjusted	1.032	0.961	1.108	0.391	1.084	0.931	1.263	0.3
	Neutrophil								
	Crude	1.004	0.859	1.173	0.963	1.078	1.000	1.163	**0.05**
	*Adjusted	1.054	0.975	1.140	0.185	0.961	0.816	1.132	0.636
VLDL (>30 mg/dL)	HP								
	Crude	1.060	0.993	1.131	0.081	1.043	0.905	1.203	0.56
	*Adjusted	1.039	0.971	1.112	0.265	1.134	0.977	1.317	0.098
	Mononuclear cell								
	Crude	1.058	0.988	1.133	0.108	1.11	0.959	1.284	0.162
	*Adjusted	1.032	0.961	1.108	0.391	1.084	0.931	1.263	0.3
	Neutrophil								
	Crude	1.078	1.000	1.163	**0.05**	1.004	0.859	1.173	0.963
	*Adjusted	1.054	0.975	1.14	0.185	0.961	0.816	1.132	0.636

HP, *Helicobacter pylori;* HDL, high-density lipoprotein; LDL, low-density lipoprotein; VLDL, very-low-density lipoprotein; *Adjusted for age, body mass index, smoking, exercise and alcohol consumption; *p* < 0.05 is shown in bold.

## Abbreviations

KBSMC	Kangbuk Samsung Medical Center
HP	*Helicobacter pylori*
CVD	Cardiovascular disease
LDL	Low-density lipoprotein
VLDL	Very-low-density lipoprotein
TG	Triglyceride
HDL	High-density lipoprotein
USS	Updated Sydney system
SBP	Systolic blood pressure
DBP	Diastolic blood pressure
BMI	Body mass index
